# Supplementary material for: Revealing undergraduate biology students’ conception of variability and error bars within graphing
Source: PLoS One. 2026 Mar 2;21(3):e0343301. doi: 10.1371/journal.pone.0343301 (PMC12952588; doi:10.1371/journal.pone.0343301)
Supplement: S2 File — (DOCX) [file pone.0343301.s002.docx]

| Category | Code | Definitiont | Examples | Buzzwords/Notes |
| --- | --- | --- | --- | --- |
| Broad terms | Variability (alone) | Use of the term(s) variation and/or variability without any context | (2590340): "The error bars provide you with the uncertainty of your measurements, along with variation between the two variables."  (2590578): "the errors bars show the variability. For the yes column, the error bars show that the mean can be with .9-1.66 with a most likely 95% confidence interval." | Vary (in the right context), variation, variability |
|  | Variability in data | Use of the term(s) variation and/or variability in regard to data, graph, chart, experimentation, section, results, etc. | (2590377): "The error bars show how much variation there is within the data."  (2391559): "error bars represent variability in that data set"  (2390637): "Error bars represent the variation of uncertainty within a data set."  (2590507): "Error bars show how much uncertainty or variation is in a data set" | Vary (in the right context), variation, variability |
|  | Uncertainty (alone) | Use of the term uncertainty without any context | (2608273): “They provide the degree of uncertainty”  (2602070): “The error bars represent the uncertainty. So for the answer yes the true value lies between 0 and 60.” |  |
|  | Uncertainty in data | Use of the term uncertainty in the context of data, graphing, chart, experimentation, values, measurement, etc. | (2443161): "Error bars show uncertainty in graphs and their values."  (2443703): "It is the uncertainty that comes from the data. It shows the difference between the two groups being examined." |  |
|  | Significance | Use of term significance/significant without purposeful reference | (2457610): "The error bars show if the data is significant or not."  (2595577): "The error bars tell you if there is a significant difference in your study aspects."  (2614906): "Error bars a way of showing statistical significance especially in bar graphs. The communicate uncertainty in the results rather than just mere bars."  (2610073): "The error bars determine whether the data is significant or insignificant."  (2561186): "The error bars show whether there is a statistical significant difference between the 2 data sets."  (2610639): "They display a type of range of the data around that point, usually a mean or some number with statistical significance." |  |
| Form of Error | Error | Error without context or with multiple contexts so its unclear what the form of error is | (2441021): "It represents the room for errors that can be possibility made."  (2605635): “To show how much error there could be and the lines show that the graph could be high or lower than what is shows.”  (2563134): “I believe that it shows the error and how much the data may vary by” | mistakes |
|  | Error in data | Describing error in data; Using chart or graph to talk about the way the data are interpreted; context of the use of error leads to reference in the data | (2389637): "Error bars provide an estimated error in the represented data"  (2440961): "error bars provide information that the data can have a certain amount of error to how its been graphed." | mistakes |
|  | Margin of error | Describe error in terms of the margin of error or room for error | (2607548): "The error bars show the margin of error (how far from accurate the data could actually be)."  (2561127): “Error bars are parts of the graph that show marginal error, you would use them to show a give or take situation, like give or take 30 lobsters in an area.” | Wiggle room, range of error |
|  | Error in Experimentation | Describing error within the experimentation of the study/data. Referring to human error. Describe error in recording of data. | (2601007): "It gives you the range of error the experiment could have."  (2590587): "Represent the variability of data and used on graphs to indicate the error or uncertainty in a reported measurement."  (2600639): "i do not know, but i'm assuming it's because of human error; undocumented variabilities within an experiment. This would calculate it and if they overlap i'm assuming the p-value would be lower and you reject null hypothesis"  (2603428): "I'm not entirely sure about my response, but I think the error bars show the biggest and lowest point on the bars, ones that might be due to errors and mistakes when recording data."  (2595943): “they tell you how much error or uncertainty is possible in your calculations once they are plotted.” | Replications, samples, experiment, sampling error, calculations, measurement, mistakes |
|  | Accuracy/Precision | Describing the error in using the terms accuracy and/or precision of the data/study/experiment | (2590891): "Error bars represent the variability of the data and how accurate the measurement is. In this case, NoFishing habitat had more spread in the data when compared to YesFishing habitat."  (2441004): "Error bars reveal the uncertainty of the data by indicating how far or precise the data is."  (2608194): "The error bars show standard deviation, where the data points mostly lie in for each option. If you were the run the experiment again identically, you would expect your values to be within the error bar."  (2606711): "That the data in the yes column is not as precise as the no data." | “How much the data may be off by”; reliability; void; talking about confidence in the data; how far off; true/actual values; true/actual data; incorrect; consistency |
| Purpose/Defining by Example | Standard Deviation | Describing error bars as being or showing standard deviation | (2590118): "The error bars provide the standard deviation."  (2590540): "I would assume it is showing either the 95% confidence interval or the standard deviations of the means." |  |
|  | Standard Error | Describing error bars as being or showing standard error  Note: Because standard error is a specific type of error, choose this and not an error code | (2590302): "The standard error of the data set." |  |
|  | Confidence Interval | Describe error bars as being/showing/representing confidence intervals | (2590540): "I would assume it is showing either the 95% confidence interval or the standard deviations of the means."  (2590578): "the errors bars show the variability. For the yes column, the error bars show that the mean can be with .9-1.66 with a most likely 95% confidence interval." | Could be talking about 95% CI |
|  | Outliers | Describe error bars as showing outliers in the data, study, and/or experiment | (2590693): "That there are outliers in the data and the error bars show the range outside of the average." |  |
|  | Distribution | Describe error bars as showing the spread in the data; Describing data form; talking about the data as a whole; how the data looks | (2590555): "the bar errors provide us with any other possible values for the two categories."  (2590891): "Error bars represent the variability of the data and how accurate the measurement is. In this case, NoFishing habitat had more spread in the data when compared to YesFishing habitat."  (2603006): "The error bars display the variability of the data. Data with error bars may experience greater variation. The data for this group may be further scattered."  (2461924): "The error bars present the additional possible values that may be viable to the study, as there may have been bias or skewed data during collection."  (2561139): "The errors bars show us the medians of the data or where most of the points in these data sets occurs, so more of the data resides in the roughly 0.8 ranges, and the top of the error bars show outliers, and this helps determine how far off the outliers were from the overall average data.  (2590566): "It gives the audience a know of how far the data skews" | Skewness, skewed, condensed, dispersed, distribution, deviation, clumped, median, high/low, above/below, “possible values”  Percentile scores |
|  | Significance Testing | Describe using the error bars to test for significance between treatments by giving context for the use of the term significant and/or significance | (2590680): "I believe error bars indicate confidence intervals, mean, staandard deviatiosn, and much more. They provide a visual view to statistical significance."  (2603850): "Because the error bars overlap greatly, we are able to assume that there is no statistical difference in means of the y-variable between the yes and no groups."  (2604901): "Error bars help readers to visualize if there is a significant difference in the data when comparing two or more things." | Hypothesis and prediction testing  Looking for a trend |
|  | Lowest and Highest points/ Range | Describe the error bars as being the lowest and highest points; Use of the term range in the right context; Describe a min/max or lowest/highest; bookend values for the error bars    upper/lower (used in the right context) | (2441991): "The error bars tell us that the data can range anywhere from the top of the error bar to the bottom of it. It is a way to show uncertainty of the data."  (2590693): "That there are outliers in the data and the error bars show the range outside of the average."  (2446817): "Error bars provide data shown as a range between the error bar. This means that there was data found in those ranges but not enough to extend the bar graph. Shows room for error in the data."  (2602222): "I do not know. But ill give it a shot, I think errors bars provides the lowest to highest point, or maybe the average of each bar."  (2590578): "the errors bars show the variability. For the yes column, the error bars show that the mean can be with .9-1.66 with a most likely 95% confidence interval."  (2608658): "The error bars provide you with the standard deviation meaning that when you read the graph, the actual true value it can be is the value you read and then plus or minus the value that the error bars tell you" |  |
| Trend Analysis | Mean/Average | Mention of bar being an average or mean | (2590298): "this is typically a t test or an anova test that helps use the same mean variables in two different populations"  (2590589): "the variability of the set of data; standard deviation (mean is shown)"  (2458650): "The error bars allow you to see how far the mean is from the standard deviation."  (2590693): "That there are outliers in the data and the error bars show the range outside of the average." |  |
|  | Comparison | Talk about difference between treatments.  Note: no discussion of overlapping error bars in the context of the comparison  Note: do not double code with hypothesis testing  Note: Reference to difference in the dataset does NOT warrant this code | (2590387): "The error bars shows that there is more variation in the "no" bar graph."  (2442603): "The smaller the error bar like in Yes shows that there was less variance in the data set. Meanwhile a larger error bar like in No means more variance and uncertainty overall."  (2605777): "The error bars are very similar between the two bars of the graph, and are very long, which tells you that there is a lot of variability of each data point from the mean of the data set"  (24437030): "It is the uncertainty that comes from the data. It shows the difference between the two groups being examined."  (2606120): "The error bars show how much variation there is in a certain set of data. For the first bar, there is a lot of variation, since the error bar is very long. The second bar still has some variation, but not as much as the first one."  (2607015): "The yes category has a large fluctuation, while the no category has less fluctuation. Thus, even though the yes category has a lower average, that may not mean much due to the amount of variability in the data itself." | Can mention one treatment without mentioning the second;  More, bigger, less than, difference, compared to, |
|  | Distance from the mean | Specifically mentioning distance from the mean as a way of explaining the error bars;  Note: Look for “from the”  Note: Do not double code with mean/average | (2604153): "The error bars show you how much variation there is within each section, as the error bars represent the standard deviation. The standard deviation represents how far certain values are from the mean, so if there is a low standard deviation, there would not be a lot of variation."  (2605732): "How much the individual data varies from the mean of each category."  (2605987): "The error bars provide the standard deviation, which is how far the data sets lie from the mean. It shows the amount of uncertainty in each data set." | Other words can be used instead of distance: in relation, range, varies, deviates |
|  | Size | Description of the size or length or height of the error bars | (2590250): "Long error bars mean that the data is less concentrated within a certain grouping of numbers. With Shorter error bars the values would be lumped closer together."  (2605147): "Mean is represented by the length of the bar and standard deviation is represented by the error bars."  (2605777): "The error bars are very similar between the two bars of the graph, and are very long, which tells you that there is a lot of variability of each data point from the mean of the data set" | If distribution is talked about in the context of size, size should be the code given; could say one error bar is greater than the other |
|  | Overlap | Discussion of whether the error bars overlap | (2446952): "The error bar shows that data for Yes and No category is overlapping in range and it is hard to conclude if data support a conclusive difference between the two categories."  (2600639): "I do not know, but I’m assuming it's because of human error; undocumented variabilities within an experiment. This would calculate it and if they overlap I’m assuming the p-value would be lower, and you reject null hypothesis"  (2604892): "The error bars provide information about the standard deviation of the data. Because the error bars overlap much within this data, shows that the data between the habitats are not statistically significant." | Match up |
|  | Statistical Test | Describes error bars can be used in conjunction with specific stats tests;  If “true mean” or “true average” are used, do not double code with mean/average above | (2590298): "this is typically a t test or an anova test that helps use the same mean variables in two different populations"  (2633959): “The error bars that the bar graph display show the standard deviation on both sides of the mean. They show that the true average could differ by that amount based on the statistics.” | Describing “true average” or “true mean”  Using true with a specific variable (true lobster density)  Talking about sample size |
| Answer Confidence | Yes | Response indicates that the student was confident in their answer |  |  |
|  | Unsure | Response indicates student was unsure of their response but provided an answer anyway |  | I think, maybe, unsure, question marks, I believe |
|  | I do not know | Writes I do not know or unsure without any other explanation |  |  |
