# Supplementary material for: Revealing undergraduate biology students’ conception of variability and error bars within graphing
Source: PLoS One. 2026 Mar 2;21(3):e0343301. doi: 10.1371/journal.pone.0343301 (PMC12952588; doi:10.1371/journal.pone.0343301)
Supplement: S3 File — (DOCX) [file pone.0343301.s003.docx]

library(ggplot2)

library(rstatix)

library(FSA)

#Checking number of codes with graph type data

data<-read.csv("GraphTypeTotals.csv")

data$Graphtype<-as.factor(data$Graphtype)

data %>% shapiro_test(Total_All)

#P value less than 0.05 so data is non normal, need to run non-parametric tests, assuming this for the other columns as well

kruskal.test(Total_All~Graphtype, data=data)

#Kruskal-Wallis chi-squared = 249.23, df = 5, p-value < 2.2e-16

Dunn<-dunnTest(Total_All~Graphtype,data=data, method="bonferroni")

Dunn

kruskal.test(Total_Broad~Graphtype, data=data)

#Kruskal-Wallis chi-squared = 50.24, df = 5, p-value = 1.238e-09

Dunn<-dunnTest(Total_Broad~Graphtype,data=data, method="bonferroni")

Dunn

kruskal.test(Total_Error~Graphtype, data=data)

#Kruskal-Wallis chi-squared = 4.4232, df = 5, p-value = 0.4902

kruskal.test(Total_Purpose~Graphtype, data=data)

#Kruskal-Wallis chi-squared = 138.9, df = 5, p-value < 2.2e-16

Dunn<-dunnTest(Total_Purpose~Graphtype,data=data, method="bonferroni")

Dunn

kruskal.test(Total_Trend~Graphtype, data=data)

#Kruskal-Wallis chi-squared = 195.7, df = 5, p-value < 2.2e-16

Dunn<-dunnTest(Total_Trend~Graphtype,data=data, method="bonferroni")

Dunn

graph1<-read.csv("Graph_1.csv")

rownames(graph1)<-graph1[,1]

graph1<-graph1[,-1]

a<-chisq.test(graph1)

a

#test stat = 142.69, df = 10, p < 0.0001

chisq.test(graph1)$stdres

alpha=0.05

alpha_adj<-alpha/(nrow(graph1)*ncol(graph1))

qnorm(alpha_adj/2)

residuals(a)

fisher.test(graph1)

fisher.test(graph1, simulate.p.value = TRUE, B=10000)

#Fisher.test pvalue = <0.0001 10e-5

graph1<-read.csv("Graph_1_graph.csv")

aa<-ggplot(graph1, aes(fill=Var_Ans, y=Number, x=Graph_made)) +

geom_bar(position="stack", stat="identity")+

theme_bw() +

theme(panel.background = element_rect(fill = "white", colour = NA),

panel.grid.minor = element_blank(),

panel.grid.major = element_blank(),

plot.background = element_rect(fill = "white", colour = NA),

panel.border = element_rect(fill = NA, colour = "black"),

text = element_text(color ="black", size = 20, family = "serif"),

axis.text.x = element_text(color ="black", size = 20, angle = 0),

axis.text.y = element_text(color ="black", size = 20, angle = 0))+

ggtitle("Graph 1")+

xlab(element_blank())+

theme(axis.title.x=element_text(angle = 0, size = 20)) +

theme(axis.text.x = element_text(angle = 90, vjust = 0.5, hjust=1))+

ylab("Frequency of Students") +

theme(axis.title.y=element_text(angle = 90, size = 20))+

theme(plot.title = element_text(hjust = 0.5))+

theme(legend.position = "right",

legend.justification = c("right", "bottom"),

legend.box.just = "right", legend.margin = margin(6, 6, 6, 6),

legend.text = element_text(size = 20, family = "serif"),

legend.title = element_blank())

aa

graph2<-read.csv("Graph_2.csv")

rownames(graph2)<-graph2[,1]

graph2<-graph2[,-1]

bb<-chisq.test(graph2)

residuals(bb)

fisher.test(graph2)

fisher.test(graph2, simulate.p.value = TRUE, B=10000)

#chi-square test stat = 37.882, df = 10, p < 0.001

#fisher.alpha pvalue = 2e-4 (0.0002)

chisq.test(graph2)$stdres

alpha=0.05

alpha_adj<-alpha/(nrow(graph2)*ncol(graph2))

qnorm(alpha_adj/2)

graph2<-read.csv("Graph_2_graph.csv")

b<-ggplot(graph2, aes(fill=Var_Ans, y=Number, x=Graph_made)) +

geom_bar(position="stack", stat="identity")+

theme_bw() +

theme(panel.background = element_rect(fill = "white", colour = NA),

panel.grid.minor = element_blank(),

panel.grid.major = element_blank(),

plot.background = element_rect(fill = "white", colour = NA),

panel.border = element_rect(fill = NA, colour = "black"),

text = element_text(color ="black", size = 20, family = "serif"),

axis.text.x = element_text(color ="black", size = 20, angle = 0),

axis.text.y = element_text(color ="black", size = 20, angle = 0))+

xlab(element_blank())+

ggtitle("Graph 2")+

theme(axis.title.x=element_text(angle = 0, size = 20)) +

theme(axis.text.x = element_text(angle = 90, vjust = 0.5, hjust=1))+

ylab("Frequency of Students") +

theme(axis.title.y=element_text(angle = 90, size = 20))+

theme(plot.title = element_text(hjust = 0.5))+

theme(legend.position = "right",

legend.justification = c("right", "bottom"),

legend.box.just = "right", legend.margin = margin(6, 6, 6, 6),

legend.text = element_text(size = 20, family = "serif"),

legend.title = element_blank())

b

graph3<-read.csv("Graph_3.csv")

rownames(graph3)<-graph3[,1]

graph3<-graph3[,-1]

cc<-chisq.test(graph3)

residuals(cc)

fisher.test(graph3, simulate.p.value = TRUE, B=10000)

#chi-square test stat = 34.516, df = 10, pvalue = 0.00015

#fisher.alpha pvalue < 0.0001

chisq.test(graph3)$stdres

alpha=0.05

alpha_adj<-alpha/(nrow(graph3)*ncol(graph3))

qnorm(alpha_adj/2)

graph3<-read.csv("Graph_3_graph.csv")

c<-ggplot(graph3, aes(fill=Var_Ans, y=Number, x=Graph_made)) +

geom_bar(position="stack", stat="identity")+

theme_bw() +

theme(panel.background = element_rect(fill = "white", colour = NA),

panel.grid.minor = element_blank(),

panel.grid.major = element_blank(),

plot.background = element_rect(fill = "white", colour = NA),

panel.border = element_rect(fill = NA, colour = "black"),

text = element_text(color ="black", size = 20, family = "serif"),

axis.text.x = element_text(color ="black", size = 20, angle = 0),

axis.text.y = element_text(color ="black", size = 20, angle = 0))+

xlab(element_blank())+

ggtitle("Graph 3")+

theme(axis.title.x=element_text(angle = 0, size = 20)) +

theme(axis.text.x = element_text(angle = 90, vjust = 0.5, hjust=1))+

ylab("Frequency of Students") +

theme(axis.title.y=element_text(angle = 90, size = 20))+

theme(plot.title = element_text(hjust = 0.5))+

theme(legend.position = "right",

legend.justification = c("right", "bottom"),

legend.box.just = "right", legend.margin = margin(6, 6, 6, 6),

legend.text = element_text(size = 20, family = "serif"),

legend.title = element_blank())

c

graph4<-read.csv("Graph_4.csv")

rownames(graph4)<-graph4[,1]

graph4<-graph4[,-1]

dd<-chisq.test(graph4)

residuals(dd)

fisher.test(graph4, simulate.p.value = TRUE, B=10000)

#chi-square test stat = 88.247, df = 10, pvalue = 1.192e-14

#did not need to run fisher.test

chisq.test(graph4)$stdres

alpha=0.05

alpha_adj<-alpha/(nrow(graph4)*ncol(graph4))

qnorm(alpha_adj/2)

graph4<-read.csv("Graph_4_graph.csv")

d<-ggplot(graph4, aes(fill=Var_Ans, y=Number, x=Graph_made)) +

geom_bar(position="stack", stat="identity")+

theme_bw() +

theme(panel.background = element_rect(fill = "white", colour = NA),

panel.grid.minor = element_blank(),

panel.grid.major = element_blank(),

plot.background = element_rect(fill = "white", colour = NA),

panel.border = element_rect(fill = NA, colour = "black"),

text = element_text(color ="black", size = 20, family = "serif"),

axis.text.x = element_text(color ="black", size = 20, angle = 0),

axis.text.y = element_text(color ="black", size = 20, angle = 0))+

xlab(element_blank())+

ggtitle("Graph 4")+

theme(axis.title.x=element_text(angle = 0, size = 20)) +

theme(axis.text.x = element_text(angle = 90, vjust = 0.5, hjust=1))+

ylab("Frequency of Students") +

theme(axis.title.y=element_text(angle = 90, size = 20))+

theme(plot.title = element_text(hjust = 0.5))+

theme(legend.position = "right",

legend.justification = c("right", "bottom"),

legend.box.just = "right", legend.margin = margin(6, 6, 6, 6),

legend.text = element_text(size = 20, family = "serif"),

legend.title = element_blank())

d

graph5<-read.csv("Graph_5.csv")

rownames(graph5)<-graph5[,1]

graph5<-graph5[,-1]

ee<-chisq.test(graph5)

residuals(ee)

fisher.test(graph5, simulate.p.value = TRUE, B=10000)

#chi-square test stat = 39.811, df = 10, pvalue = 1.829e-5

#fisher test p value = 2e-4

chisq.test(graph5)$stdres

alpha=0.05

alpha_adj<-alpha/(nrow(graph5)*ncol(graph5))

qnorm(alpha_adj/2)

graph5<-read.csv("Graph_5_graph.csv")

e<-ggplot(graph5, aes(fill=Var_Ans, y=Number, x=Graph_made)) +

geom_bar(position="stack", stat="identity")+

theme_bw() +

theme(panel.background = element_rect(fill = "white", colour = NA),

panel.grid.minor = element_blank(),

panel.grid.major = element_blank(),

plot.background = element_rect(fill = "white", colour = NA),

panel.border = element_rect(fill = NA, colour = "black"),

text = element_text(color ="black", size = 20, family = "serif"),

axis.text.x = element_text(color ="black", size = 20, angle = 0),

axis.text.y = element_text(color ="black", size = 20, angle = 0))+

xlab(element_blank())+

ggtitle("Graph 5")+

theme(axis.title.x=element_text(angle = 0, size = 20)) +

theme(axis.text.x = element_text(angle = 90, vjust = 0.5, hjust=1))+

ylab("Frequency of Students") +

theme(axis.title.y=element_text(angle = 90, size = 20))+

theme(plot.title = element_text(hjust = 0.5))+

theme(legend.position = "right",

legend.justification = c("right", "bottom"),

legend.box.just = "right", legend.margin = margin(6, 6, 6, 6),

legend.text = element_text(size = 20, family = "serif"),

legend.title = element_blank())

e

graph6<-read.csv("Graph_6.csv")

rownames(graph6)<-graph6[,1]

graph6<-graph6[,-1]

ff<-chisq.test(graph6)

residuals(ff)

fisher.test(graph6, simulate.p.value = TRUE, B=10000)

#chi-square test stat = 49.717, df = 10, pvalue = 3.008e-7

#did not need to run fisher.test

chisq.test(graph6)$stdres

alpha=0.05

alpha_adj<-alpha/(nrow(graph6)*ncol(graph6))

qnorm(alpha_adj/2)

graph6<-read.csv("Graph_6_graph.csv")

f<-ggplot(graph6, aes(fill=Var_Ans, y=Number, x=Graph_made)) +

geom_bar(position="stack", stat="identity")+

theme_bw() +

theme(panel.background = element_rect(fill = "white", colour = NA),

panel.grid.minor = element_blank(),

panel.grid.major = element_blank(),

plot.background = element_rect(fill = "white", colour = NA),

panel.border = element_rect(fill = NA, colour = "black"),

text = element_text(color ="black", size = 20, family = "serif"),

axis.text.x = element_text(color ="black", size = 20, angle = 0),

axis.text.y = element_text(color ="black", size = 20, angle = 0))+

xlab(element_blank())+

ggtitle("Graph 6")+

theme(axis.title.x=element_text(angle = 0, size = 20)) +

theme(axis.text.x = element_text(angle = 90, vjust = 0.5, hjust=1))+

ylab("Frequency of Students") +

theme(axis.title.y=element_text(angle = 90, size = 20))+

theme(plot.title = element_text(hjust = 0.5))+

theme(legend.position = "right",

legend.justification = c("right", "bottom"),

legend.box.just = "right", legend.margin = margin(6, 6, 6, 6),

legend.text = element_text(size = 20, family = "serif"),

legend.title = element_blank())

f

library(cowplot)

bottom_row <- plot_grid(e,f,

labels = c('e', 'f'), label_size=24, label_fontfamily = "sans",

label_fontface = "plain", align = "v", axis = "t", rel_widths = c(1), ncol = 2, nrow = 1)

middle_row <- plot_grid(c,d,

labels = c('c', 'd'), label_size=24, label_fontfamily = "sans",

label_fontface = "plain", align = "v", axis = "t", rel_widths = c(1), ncol = 2, nrow = 1)

upper_row <- plot_grid(aa,b,

labels = c('a', 'b'), label_size=24, label_fontfamily = "sans",

label_fontface = "plain", align = "v", axis = "t", rel_widths = c(1), ncol = 2, nrow = 1)

beta2 <- plot_grid(upper_row, middle_row, bottom_row,

label_fontfamily = "sans", rel_widths = c(1), ncol = 1)

beta2

ggsave(plot = beta2, "UpdatedDropdownbyGraph.jpg", width = 15, height = 20, dpi = 600)

maincats<-read.csv("main_categories.csv")

rownames(maincats)<-maincats[,1]

maincats<-maincats[,-1]

ww<-chisq.test(maincats)

residuals(ww)

fisher.test(maincats, simulate.p.value = TRUE, B=10000)

#chi-square test stat = 64.747, df = 15, p value = 3.78e-8

#did not need fisher.test

chisq.test(maincats)$stdres

alpha=0.05

alpha_adj<-alpha/(nrow(maincats)*ncol(maincats))

qnorm(alpha_adj/2)

maincats<-read.csv("Categories_graph.csv")

w<-ggplot(maincats, aes(fill=Code, y=Number, x=Graph_made)) +

geom_bar(position="stack", stat="identity")+

theme_bw() +

theme(panel.background = element_rect(fill = "white", colour = NA),

panel.grid.minor = element_blank(),

panel.grid.major = element_blank(),

plot.background = element_rect(fill = "white", colour = NA),

panel.border = element_rect(fill = NA, colour = "black"),

text = element_text(color ="black", size = 20, family = "serif"),

axis.text.x = element_text(color ="black", size = 20, angle = 0),

axis.text.y = element_text(color ="black", size = 20, angle = 0))+

xlab(element_blank())+

ggtitle("Categories")+

theme(axis.title.x=element_text(angle = 0, size = 20)) +

theme(axis.text.x = element_text(angle = 90, vjust = 0.5, hjust=1))+

ylab("Frequency of codes") +

theme(axis.title.y=element_text(angle = 90, size = 20))+

theme(plot.title = element_text(hjust = 0.5))+

theme(legend.position = "right",

legend.justification = c("right", "bottom"),

legend.box.just = "right", legend.margin = margin(6, 6, 6, 6),

legend.text = element_text(size = 20, family = "serif"),

legend.title = element_blank())

w

encompass<-read.csv("all_encompassing2.csv")

rownames(encompass)<-encompass[,1]

encompass<-encompass[,-1]

tt<-chisq.test(encompass)

residuals(tt)

fisher.test(encompass, simulate.p.value = TRUE, B=10000)

#Chi-square test stat = 17.949, df = 20 pvalue = 0.0.5908

#fisher.test pvalue = 0.6208

chisq.test(encompass)$stdres

alpha=0.05

alpha_adj<-alpha/(nrow(encompass)*ncol(encompass))

qnorm(alpha_adj/2)

encompass<-read.csv("all_encompassing_graph.csv")

x<-ggplot(encompass, aes(fill=Code, y=Number, x=Graph_made)) +

geom_bar(position="stack", stat="identity")+

theme_bw() +

theme(panel.background = element_rect(fill = "white", colour = NA),

panel.grid.minor = element_blank(),

panel.grid.major = element_blank(),

plot.background = element_rect(fill = "white", colour = NA),

panel.border = element_rect(fill = NA, colour = "black"),

text = element_text(color ="black", size = 20, family = "serif"),

axis.text.x = element_text(color ="black", size = 20, angle = 0),

axis.text.y = element_text(color ="black", size = 20, angle = 0))+

xlab(element_blank())+

ggtitle("Broad Terms")+

theme(axis.title.x=element_text(angle = 0, size = 20)) +

theme(axis.text.x = element_text(angle = 90, vjust = 0.5, hjust=1))+

ylab("Frequency of Students") +

theme(axis.title.y=element_text(angle = 90, size = 20))+

theme(plot.title = element_text(hjust = 0.5))+

theme(legend.position = "right",

legend.justification = c("right", "bottom"),

legend.box.just = "right", legend.margin = margin(6, 6, 6, 6),

legend.text = element_text(size = 20, family = "serif"),

legend.title = element_blank())

x

error<-read.csv("error.csv")

rownames(error)<-error[,1]

error<-error[,-1]

yy<-chisq.test(error)

residuals(yy)

fisher.test(error, simulate.p.value = TRUE, B=10000)

#chi-square test stat = 48.732 df = 20 pvalue = 0.0003

#fisher.test pvalue = 0.0002

chisq.test(error)$stdres

alpha=0.05

alpha_adj<-alpha/(nrow(error)*ncol(error))

qnorm(alpha_adj/2)

error<-read.csv("Error_graph.csv")

e<-ggplot(error, aes(fill=Code, y=Number, x=Graph_made)) +

geom_bar(position="stack", stat="identity")+

theme_bw() +

theme(panel.background = element_rect(fill = "white", colour = NA),

panel.grid.minor = element_blank(),

panel.grid.major = element_blank(),

plot.background = element_rect(fill = "white", colour = NA),

panel.border = element_rect(fill = NA, colour = "black"),

text = element_text(color ="black", size = 20, family = "serif"),

axis.text.x = element_text(color ="black", size = 20, angle = 0),

axis.text.y = element_text(color ="black", size = 20, angle = 0))+

xlab(element_blank())+

ggtitle("Error")+

theme(axis.title.x=element_text(angle = 0, size = 20)) +

theme(axis.text.x = element_text(angle = 90, vjust = 0.5, hjust=1))+

ylab("Frequency of Students") +

theme(axis.title.y=element_text(angle = 90, size = 20))+

theme(plot.title = element_text(hjust = 0.5))+

theme(legend.position = "right",

legend.justification = c("right", "bottom"),

legend.box.just = "right", legend.margin = margin(6, 6, 6, 6),

legend.text = element_text(size = 20, family = "serif"),

legend.title = element_blank())

e

purpose<-read.csv("purpose.csv")

rownames(purpose)<-purpose[,1]

purpose<-purpose[,-1]

oo<-chisq.test(purpose)

residuals(oo)

fisher.test(purpose, simulate.p.value = TRUE, B=10000)

#chi-square test stat = 61.688, df = 30, pvalue = 0.00057

#fisher.test pvalue = 0.0007

chisq.test(purpose)$stdres

alpha=0.05

alpha_adj<-alpha/(nrow(purpose)*ncol(purpose))

qnorm(alpha_adj/2)

purpose<-read.csv("Purpose_graph.csv")

z<-ggplot(purpose, aes(fill=Code, y=Number, x=Graph_made)) +

geom_bar(position="stack", stat="identity")+

theme_bw() +

theme(panel.background = element_rect(fill = "white", colour = NA),

panel.grid.minor = element_blank(),

panel.grid.major = element_blank(),

plot.background = element_rect(fill = "white", colour = NA),

panel.border = element_rect(fill = NA, colour = "black"),

text = element_text(color ="black", size = 20, family = "serif"),

axis.text.x = element_text(color ="black", size = 20, angle = 0),

axis.text.y = element_text(color ="black", size = 20, angle = 0))+

xlab(element_blank())+

ggtitle("Purpose")+

theme(axis.title.x=element_text(angle = 0, size = 20)) +

theme(axis.text.x = element_text(angle = 90, vjust = 0.5, hjust=1))+

ylab("Frequency of Students") +

theme(axis.title.y=element_text(angle = 90, size = 20))+

theme(plot.title = element_text(hjust = 0.5))+

theme(legend.position = "right",

legend.justification = c("right", "bottom"),

legend.box.just = "right", legend.margin = margin(6, 6, 6, 6),

legend.text = element_text(size = 20, family = "serif"),

legend.title = element_blank())

z

trend<-read.csv("trend_analysis.csv")

rownames(trend)<-trend[,1]

trend<-trend[,-1]

nn<-chisq.test(trend)

nn

residuals(nn)

fisher.test(trend, simulate.p.value = TRUE, B=10000)

#chi-square test stat = 30.942, df = 25, pvalue = 0.191

#fisher.test pvalue = 0.169

chisq.test(trend)$stdres

alpha=0.05

alpha_adj<-alpha/(nrow(trend)*ncol(trend))

qnorm(alpha_adj/2)

trend<-read.csv("Trend_graph.csv")

t<-ggplot(trend, aes(fill=Code, y=Number, x=Graph_made)) +

geom_bar(position="stack", stat="identity")+

theme_bw() +

theme(panel.background = element_rect(fill = "white", colour = NA),

panel.grid.minor = element_blank(),

panel.grid.major = element_blank(),

plot.background = element_rect(fill = "white", colour = NA),

panel.border = element_rect(fill = NA, colour = "black"),

text = element_text(color ="black", size = 20, family = "serif"),

axis.text.x = element_text(color ="black", size = 20, angle = 0),

axis.text.y = element_text(color ="black", size = 20, angle = 0))+

xlab(element_blank())+

ggtitle("Trend and Analysis")+

theme(axis.title.x=element_text(angle = 0, size = 20)) +

theme(axis.text.x = element_text(angle = 90, vjust = 0.5, hjust=1))+

ylab("Frequency of Students") +

theme(axis.title.y=element_text(angle = 90, size = 20))+

theme(plot.title = element_text(hjust = 0.5))+

theme(legend.position = "right",

legend.justification = c("right", "bottom"),

legend.box.just = "right", legend.margin = margin(6, 6, 6, 6),

legend.text = element_text(size = 20, family = "serif"),

legend.title = element_blank())

t

#Checking other categories for graph type data

OtherCats<-read.csv("GraphTypeOther.csv")

rownames(OtherCats)<-OtherCats[,1]

OtherCats<-OtherCats[,-1]

b<-chisq.test(OtherCats)

b

#X-squared = 250.42, df = 70, p-value < 2.2e-16

residuals(b)

fisher.test(OtherCats, simulate.p.value = TRUE, B=10000)

#p value < 0.0001

chisq.test(OtherCats)$stdres

alpha=0.05

alpha_adj<-alpha/(nrow(OtherCats)*ncol(OtherCats))

qnorm(alpha_adj/2)

GraphOther<-read.csv("GraphTypeOther_Graph.csv")

o<-ggplot(GraphOther, aes(fill=Code, y=Number, x=Graph_made)) +

geom_bar(position="stack", stat="identity")+

theme_bw() +

theme(panel.background = element_rect(fill = "white", colour = NA),

panel.grid.minor = element_blank(),

panel.grid.major = element_blank(),

plot.background = element_rect(fill = "white", colour = NA),

panel.border = element_rect(fill = NA, colour = "black"),

text = element_text(color ="black", size = 20, family = "serif"),

axis.text.x = element_text(color ="black", size = 20, angle = 0),

axis.text.y = element_text(color ="black", size = 20, angle = 0))+

xlab(element_blank())+

ggtitle("Combinations of Main Categories")+

theme(axis.title.x=element_text(angle = 0, size = 20)) +

theme(axis.text.x = element_text(angle = 90, vjust = 0.5, hjust=1))+

ylab("Frequency of Students") +

theme(axis.title.y=element_text(angle = 90, size = 20))+

theme(plot.title = element_text(hjust = 0.5))+

theme(legend.position = "right",

legend.justification = c("right", "bottom"),

legend.box.just = "right", legend.margin = margin(6, 6, 6, 6),

legend.text = element_text(size = 20, family = "serif"),

legend.title = element_blank())

o

bottom_row <- plot_grid(z,t,

labels = c('e', 'f'), label_size=24, label_fontfamily = "sans",

label_fontface = "plain", align = "v", axis = "t", rel_widths = c(1), ncol = 2, nrow = 1)

middle_row <- plot_grid(x,e,

labels = c('c', 'd'), label_size=24, label_fontfamily = "sans",

label_fontface = "plain", align = "v", axis = "t", rel_widths = c(1), ncol = 2, nrow = 1)

upper_row <- plot_grid(w,o,

labels = c('a', 'b'), label_size=24, label_fontfamily = "sans",

label_fontface = "plain", align = "v", axis = "t", rel_widths = c(1), ncol = 2, nrow = 1)

beta2 <- plot_grid(upper_row, middle_row, bottom_row,

label_fontfamily = "sans", rel_widths = c(1), ncol = 1)

beta2

ggsave(plot = beta2, "UpdatedCodesbyGraph.jpg", width = 15, height = 20, dpi = 600)

answer<-read.csv("Answers_by_graph.csv")

rownames(answer)<-answer[,1]

answer<-answer[,-1]

mm<-chisq.test(answer)

residuals(mm)

fisher.test(answer, simulate.p.value = TRUE, B=10000)

#chi-square test stat = 102.49, df = 10, p value = 2.2e-16

#fisher.test = <0.0001

chisq.test(answer)$stdres

alpha=0.05

alpha_adj<-alpha/(nrow(answer)*ncol(answer))

qnorm(alpha_adj/2)

answer<-read.csv("answering_graph.csv")

ag<-ggplot(answer, aes(fill=Code, y=Number, x=Graph_made)) +

geom_bar(position="stack", stat="identity")+

theme_bw() +

theme(panel.background = element_rect(fill = "white", colour = NA),

panel.grid.minor = element_blank(),

panel.grid.major = element_blank(),

plot.background = element_rect(fill = "white", colour = NA),

panel.border = element_rect(fill = NA, colour = "black"),

text = element_text(color ="black", size = 20, family = "serif"),

axis.text.x = element_text(color ="black", size = 20, angle = 0),

axis.text.y = element_text(color ="black", size = 20, angle = 0))+

xlab(element_blank())+

ggtitle("Graph Made")+

theme(axis.title.x=element_text(angle = 0, size = 20)) +

theme(axis.text.x = element_text(angle = 90, vjust = 0.5, hjust=1))+

ylab("Frequency of Students") +

theme(axis.title.y=element_text(angle = 90, size = 20))+

theme(plot.title = element_text(hjust = 0.5))+

theme(legend.position = "right",

legend.justification = c("right", "bottom"),

legend.box.just = "right", legend.margin = margin(6, 6, 6, 6),

legend.text = element_text(size = 20, family = "serif"),

legend.title = element_blank())

ag

#Student year

Confidence<-read.csv("student year table 3.csv")

rownames(Confidence)<-Confidence[,1]

Confidence<-Confidence[,-1]

GraphbyYear<-read.csv("GraphByYear.csv")

rownames(GraphbyYear)<-GraphbyYear[,1]

GraphbyYear<-GraphbyYear[,-1]

c<-chisq.test(Confidence)

#X-squared = 21.5, df = 6, p-value = 0.001491

residuals(c)

residuals(b)

chisq.test(Confidence)$stdres

alpha=0.05

alpha_adj<-alpha/(nrow(Confidence)*ncol(Confidence))

qnorm(alpha_adj/2)

Answers.y<-read.csv("answers_year.csv")

ay<-ggplot(Answers.y, aes(fill=Code, y=Number, x=fct_inorder(Year))) +

geom_bar(position="stack", stat="identity")+

theme_bw() +

theme(panel.background = element_rect(fill = "white", colour = NA),

panel.grid.minor = element_blank(),

panel.grid.major = element_blank(),

plot.background = element_rect(fill = "white", colour = NA),

panel.border = element_rect(fill = NA, colour = "black"),

text = element_text(color ="black", size = 20, family = "serif"),

axis.text.x = element_text(color ="black", size = 20, angle = 0),

axis.text.y = element_text(color ="black", size = 20, angle = 0))+

xlab(element_blank())+

ggtitle("Student year")+

theme(axis.title.x=element_text(angle = 0, size = 20)) +

theme(axis.text.x = element_text(angle = 90, vjust = 0.5, hjust=1))+

ylab("Frequency of Students") +

theme(axis.title.y=element_text(angle = 90, size = 20))+

theme(plot.title = element_text(hjust = 0.5))+

theme(legend.position = "right",

legend.justification = c("right", "bottom"),

legend.box.just = "right", legend.margin = margin(6, 6, 6, 6),

legend.text = element_text(size = 20, family = "serif"),

legend.title = element_blank())

ay

d<-chisq.test(GraphbyYear)

d

#X-squared = 92.602, df = 15, p-value = 3.24e-13

chisq.test(GraphbyYear)$stdres

alpha=0.05

alpha_adj<-alpha/(nrow(GraphbyYear)*ncol(GraphbyYear))

qnorm(alpha_adj/2)

graphmade.y<-read.csv("graphmadebyyear_graph.csv")

gy<-ggplot(graphmade.y, aes(fill=Year, y=Number, x=Code)) +

geom_bar(position="stack", stat="identity")+

theme_bw() +

theme(panel.background = element_rect(fill = "white", colour = NA),

panel.grid.minor = element_blank(),

panel.grid.major = element_blank(),

plot.background = element_rect(fill = "white", colour = NA),

panel.border = element_rect(fill = NA, colour = "black"),

text = element_text(color ="black", size = 20, family = "serif"),

axis.text.x = element_text(color ="black", size = 20, angle = 0),

axis.text.y = element_text(color ="black", size = 20, angle = 0))+

xlab(element_blank())+

ggtitle("Student year")+

theme(axis.title.x=element_text(angle = 0, size = 20)) +

theme(axis.text.x = element_text(angle = 90, vjust = 0.5, hjust=1))+

ylab("Frequency of Students") +

theme(axis.title.y=element_text(angle = 90, size = 20))+

theme(plot.title = element_text(hjust = 0.5))+

theme(legend.position = "right",

legend.justification = c("right", "bottom"),

legend.box.just = "right", legend.margin = margin(6, 6, 6, 6),

legend.text = element_text(size = 20, family = "serif"),

legend.title = element_blank())+

scale_y_continuous(limits = c(0,1), breaks = c(0.25,0.5,0.75,1))

gy

#Majors

Confidence<-read.csv("Major table 3.csv")

rownames(Confidence)<-Confidence[,1]

Confidence<-Confidence[,-1]

GraphbyMajor<-read.csv("GraphByMajor.csv")

rownames(GraphbyMajor)<-GraphbyMajor[,1]

GraphbyMajor<-GraphbyMajor[,-1]

c<-chisq.test(Confidence)

#X-squared = 107.18, df = 6, p-value < 2.2e-16

residuals(c)

chisq.test(Confidence)$stdres

alpha=0.05

alpha_adj<-alpha/(nrow(Confidence)*ncol(Confidence))

qnorm(alpha_adj/2)

Answers.m<-read.csv("answers_major.csv")

am<-ggplot(Answers.m, aes(fill=Code, y=Number, x=fct_inorder(Major))) +

geom_bar(position="stack", stat="identity")+

theme_bw() +

theme(panel.background = element_rect(fill = "white", colour = NA),

panel.grid.minor = element_blank(),

panel.grid.major = element_blank(),

plot.background = element_rect(fill = "white", colour = NA),

panel.border = element_rect(fill = NA, colour = "black"),

text = element_text(color ="black", size = 20, family = "serif"),

axis.text.x = element_text(color ="black", size = 20, angle = 0),

axis.text.y = element_text(color ="black", size = 20, angle = 0))+

xlab(element_blank())+

ggtitle("Major")+

theme(axis.title.x=element_text(angle = 0, size = 20)) +

theme(axis.text.x = element_text(angle = 90, vjust = 0.5, hjust=1))+

ylab("Frequency of Students") +

theme(axis.title.y=element_text(angle = 90, size = 20))+

theme(plot.title = element_text(hjust = 0.5))+

theme(legend.position = "right",

legend.justification = c("right", "bottom"),

legend.box.just = "right", legend.margin = margin(6, 6, 6, 6),

legend.text = element_text(size = 20, family = "serif"),

legend.title = element_blank())

am

beta2 <- plot_grid(ag, ay, am, labels = c('a', 'b', 'c'), label_size = 24,

label_fontfamily = "sans", rel_widths = c(1), ncol = 1)

beta2

ggsave(plot = beta2, "UpdatedConfidenceGraphs.jpg", width = 10, height = 27, dpi = 600)

j<-chisq.test(GraphbyMajor)

j

#X-squared = 55.981, df = 15, p-value 1.22e-6

chisq.test(GraphbyMajor)$stdres

alpha=0.05

alpha_adj<-alpha/(nrow(GraphbyMajor)*ncol(GraphbyMajor))

qnorm(alpha_adj/2)

graphmade<-read.csv("graphmadebymajor_graph.csv")

gm<-ggplot(graphmade, aes(fill=Code, y=Number, x=Graph_made)) +

geom_bar(position="stack", stat="identity")+

theme_bw() +

theme(panel.background = element_rect(fill = "white", colour = NA),

panel.grid.minor = element_blank(),

panel.grid.major = element_blank(),

plot.background = element_rect(fill = "white", colour = NA),

panel.border = element_rect(fill = NA, colour = "black"),

text = element_text(color ="black", size = 20, family = "serif"),

axis.text.x = element_text(color ="black", size = 20, angle = 0),

axis.text.y = element_text(color ="black", size = 20, angle = 0))+

xlab(element_blank())+

ggtitle("Major")+

theme(axis.title.x=element_text(angle = 0, size = 20)) +

theme(axis.text.x = element_text(angle = 90, vjust = 0.5, hjust=1))+

ylab("Frequency of Students") +

theme(axis.title.y=element_text(angle = 90, size = 20))+

theme(plot.title = element_text(hjust = 0.5))+

theme(legend.position = "right",

legend.justification = c("right", "bottom"),

legend.box.just = "right", legend.margin = margin(6, 6, 6, 6),

legend.text = element_text(size = 20, family = "serif"),

legend.title = element_blank())+

scale_y_continuous(limits = c(0,1), breaks = c(0.25,0.5,0.75,1))

gm

#Course Type

graphmade.c<-read.csv("graphmadebycourse_graph.csv")

gc<-ggplot(graphmade.c, aes(fill=Code, y=Number, x=Graph_made)) +

geom_bar(position="stack", stat="identity")+

theme_bw() +

theme(panel.background = element_rect(fill = "white", colour = NA),

panel.grid.minor = element_blank(),

panel.grid.major = element_blank(),

plot.background = element_rect(fill = "white", colour = NA),

panel.border = element_rect(fill = NA, colour = "black"),

text = element_text(color ="black", size = 20, family = "serif"),

axis.text.x = element_text(color ="black", size = 20, angle = 0),

axis.text.y = element_text(color ="black", size = 20, angle = 0))+

xlab(element_blank())+

ggtitle("Course Type")+

theme(axis.title.x=element_text(angle = 0, size = 20)) +

theme(axis.text.x = element_text(angle = 90, vjust = 0.5, hjust=1))+

ylab("Frequency of Students") +

theme(axis.title.y=element_text(angle = 90, size = 20))+

theme(plot.title = element_text(hjust = 0.5))+

theme(legend.position = "right",

legend.justification = c("right", "bottom"),

legend.box.just = "right", legend.margin = margin(6, 6, 6, 6),

legend.text = element_text(size = 20, family = "serif"),

legend.title = element_blank())+

scale_y_continuous(limits = c(0,1), breaks = c(0.25,0.5,0.75,1))

gc

beta2 <- plot_grid(gy, gm, gc, labels = c('a', 'b', 'c'), label_size = 24,

label_fontfamily = "sans", rel_widths = c(1), ncol = 1)

beta2

ggsave(plot = beta2, "UpdatedDemographic.jpg", width = 10, height = 27, dpi = 600)

#making lollipop graphs

data<-read.csv("CodeLollipop.csv")

Purpose<-ggplot(data, aes(x = reorder(Purpose, -Purpose_Percent), y = Purpose_Percent)) +

geom_segment(aes(x = reorder(Purpose, -Purpose_Percent),

xend = reorder(Purpose, -Purpose_Percent),

y = 0, yend = Purpose_Percent),

color = "gray", lwd = 1) +

geom_point(size = 4, pch = 21, bg = 4, col = 1) +

xlab("Codes") +

ylab("Code Percentage") +

ggtitle("Purpose Codes")+

coord_flip() +

theme_bw() +

theme(panel.background = element_rect(fill = "white", colour = NA),

panel.grid.minor = element_blank(),

panel.grid.major = element_blank(),

plot.background = element_rect(fill = "white", colour = NA),

panel.border = element_rect(fill = NA, colour = "black"),

text = element_text(color ="black", size = 14, family = "sans"),

axis.text.x = element_text(color ="black", size = 14, angle = 0),

axis.text.y = element_text(color ="black", size = 14, angle = 0),

plot.title = element_text(hjust = 0.5))+

scale_y_continuous(limits = c(0,30), breaks = c(0,10,20,30))

Purpose

data<-read.csv("LollipopBroad_Error.csv")

Broad<-ggplot(data, aes(x = reorder(Broad, -Broad_Percent), y = Broad_Percent)) +

geom_segment(aes(x = reorder(Broad, -Broad_Percent),

xend = reorder(Broad, -Broad_Percent),

y = 0, yend = Broad_Percent),

color = "gray", lwd = 1) +

geom_point(size = 4, pch = 21, bg = 4, col = 1) +

xlab("Codes") +

ylab("Code Percentage") +

ggtitle("Broad Codes")+

coord_flip() +

theme_bw() +

theme(panel.background = element_rect(fill = "white", colour = NA),

panel.grid.minor = element_blank(),

panel.grid.major = element_blank(),

plot.background = element_rect(fill = "white", colour = NA),

panel.border = element_rect(fill = NA, colour = "black"),

text = element_text(color ="black", size = 14, family = "sans"),

axis.text.x = element_text(color ="black", size = 14, angle = 0),

axis.text.y = element_text(color ="black", size = 14, angle = 0),

plot.title = element_text(hjust = 0.5))+

scale_y_continuous(limits = c(0,50), breaks = c(0,10,20,30,40,50))

Broad

Error<-ggplot(data, aes(x = reorder(Error, -Error_Percent), y = Error_Percent)) +

geom_segment(aes(x = reorder(Error, -Error_Percent),

xend = reorder(Error, -Error_Percent),

y = 0, yend = Error_Percent),

color = "gray", lwd = 1) +

geom_point(size = 4, pch = 21, bg = 4, col = 1) +

xlab("Codes") +

ylab("Code Percentage") +

ggtitle("Error Codes")+

coord_flip() +

theme_bw() +

theme(panel.background = element_rect(fill = "white", colour = NA),

panel.grid.minor = element_blank(),

panel.grid.major = element_blank(),

plot.background = element_rect(fill = "white", colour = NA),

panel.border = element_rect(fill = NA, colour = "black"),

text = element_text(color ="black", size = 14, family = "sans"),

axis.text.x = element_text(color ="black", size = 14, angle = 0),

axis.text.y = element_text(color ="black", size = 14, angle = 0),

plot.title = element_text(hjust = 0.5))+

scale_y_continuous(limits = c(0,40), breaks = c(0,10,20,30,40))

Error

data<-read.csv("LollipopTrend.csv")

Trend<-ggplot(data, aes(x = reorder(Trend, -Trend_Percent), y = Trend_Percent)) +

geom_segment(aes(x = reorder(Trend, -Trend_Percent),

xend = reorder(Trend, -Trend_Percent),

y = 0, yend = Trend_Percent),

color = "gray", lwd = 1) +

geom_point(size = 4, pch = 21, bg = 4, col = 1) +

xlab("Codes") +

ylab("Code Percentage") +

ggtitle("Trend Codes")+

coord_flip() +

theme_bw() +

theme(panel.background = element_rect(fill = "white", colour = NA),

panel.grid.minor = element_blank(),

panel.grid.major = element_blank(),

plot.background = element_rect(fill = "white", colour = NA),

panel.border = element_rect(fill = NA, colour = "black"),

text = element_text(color ="black", size = 14, family = "sans"),

axis.text.x = element_text(color ="black", size = 14, angle = 0),

axis.text.y = element_text(color ="black", size = 14, angle = 0),

plot.title = element_text(hjust = 0.5))+

scale_y_continuous(limits = c(0,45), breaks = c(0,5,15,25,35,45))

Trend

data<-read.csv("LollipopGroups.csv")

Groups<-ggplot(data, aes(x = reorder(Group, -Percent), y = Percent)) +

geom_segment(aes(x = reorder(Group, -Percent),

xend = reorder(Group, -Percent),

y = 0, yend = Percent),

color = "gray", lwd = 1) +

geom_point(size = 4, pch = 21, bg = 4, col = 1) +

xlab("Categories") +

ylab("Code Percentage") +

ggtitle("Code Categories")+

coord_flip() +

theme_bw() +

theme(panel.background = element_rect(fill = "white", colour = NA),

panel.grid.minor = element_blank(),

panel.grid.major = element_blank(),

plot.background = element_rect(fill = "white", colour = NA),

panel.border = element_rect(fill = NA, colour = "black"),

text = element_text(color ="black", size = 14, family = "sans"),

axis.text.x = element_text(color ="black", size = 14, angle = 0),

axis.text.y = element_text(color ="black", size = 14, angle = 0),

plot.title = element_text(hjust = 0.5))+

scale_y_continuous(limits = c(0,35), breaks = c(0,5,15,25,35))

Groups

library(cowplot)

bottom_row <- plot_grid(Purpose,Trend,

labels = c('d', 'e'), label_size=16, label_fontfamily = "sans",

label_fontface = "plain", align = "v", axis = "t", rel_widths = c(1), ncol = 2, nrow = 1)

upper_row <- plot_grid(Broad,Error,

labels = c('b', 'c'), label_size=16, label_fontfamily = "sans",

label_fontface = "plain", align = "v", axis = "t", rel_widths = c(1), ncol = 2, nrow = 1)

beta2 <- plot_grid(Groups, upper_row, bottom_row, labels = c('a', ''), label_size = 16,

label_fontfamily = "serif", rel_widths = c(1), ncol = 1)

beta2

ggsave(plot = beta2, "LollipopFigureUpdated.jpg", width = 15, height = 15, dpi = 600)
